# Glutamate- and GABA-Modulated Connectivity in Auditory Hallucinations—A Combined Resting State fMRI and MR Spectroscopy Study

**DOI:** 10.3389/fpsyt.2021.643564

**Published:** 2021-02-17

**Authors:** Sarah Weber, Helene Hjelmervik, Alexander R. Craven, Erik Johnsen, Rune A. Kroken, Else-Marie Løberg, Lars Ersland, Kristiina Kompus, Kenneth Hugdahl

**Affiliations:** ^1^Department of Biological and Medical Psychology, University of Bergen, Bergen, Norway; ^2^Division of Psychiatry and NORMENT Centre of Excellence, Haukeland University Hospital, Bergen, Norway; ^3^School of Health Sciences, Kristiania University College, Bergen, Norway; ^4^Department of Clinical Engineering, Haukeland University Hospital, Bergen, Norway; ^5^Department of Clinical Medicine, University of Bergen, Bergen, Norway; ^6^Department of Addiction Medicine, Haukeland University Hospital, Bergen, Norway; ^7^Department of Clinical Psychology, University of Bergen, Bergen, Norway; ^8^Institute of Psychology, University of Tartu, Tartu, Estonia; ^9^Department of Radiology, Haukeland University Hospital, Bergen, Norway

**Keywords:** schizophrenia, psychosis, neurochemistry, neurotransmitters, functional connectivity, neuroimaging

## Abstract

**Background:** Auditory verbal hallucinations (AVH) have been linked to aberrant interhemispheric connectivity between the left and the right superior temporal gyrus (STG), labeled the interhemispheric miscommunication theory. The present study investigated if interhemispheric miscommunication is modulated at the neurochemical level by glutamate (Glu) and gamma-aminobutyric acid (GABA) concentrations in temporal and prefrontal lobe areas, as proposed by the theory.

**Methods:** We combined resting-state fMRI connectivity with MR spectroscopy (MRS) in a sample of 81 psychosis patients, comparing patients with high hallucination severity (high-AVH) and low hallucination severity (low-AVH) groups. Glu and GABA concentrations were acquired from the left STG and the anterior cingulate cortex (ACC), an area of cognitive control that has been proposed to modulate STG functioning in AVH.

**Results:** Functional connectivity showed significant interaction effects between AVH Group and ACC-recorded Glu and GABA metabolites. Follow-up tests showed that there was a significant positive association for Glu concentration and interhemispheric STG connectivity in the high-AVH group, while there was a significant negative association for GABA concentration and interhemispheric STG connectivity in the low-AVH group.

**Conclusion:** The results show neurochemical modulation of STG interhemispheric connectivity, as predicted by the interhemispheric miscommunication hypothesis. Furthermore, the findings are in line with an excitatory/inhibitory imbalance model for AVH. By combining different neuroimaging modalities, the current results provide a more comprehensive insight into the neural correlates of AVH.

## Introduction

Auditory verbal hallucinations (AVH) have been associated with aberrant functioning of the left superior temporal gyrus (STG), a brain area associated with auditory processing and speech perception (1–3). Concurrent evidence from diffusion-tensor imaging (DTI), functional magnetic resonance imaging (fMRI) and electrophysiology (EEG) shows in particular abnormal connectivity between the left STG and its right-hemispheric homolog in patients with AVH [e.g., (4–7)]. Findings of hyper-connectivity are thought to reflect an overactive positive feedback-loop where the left and the right STG continuously activate each other, causing abnormalities of auditory processing (8, 9). Recently, these findings have been summarized in the interhemispheric miscommunication theory of AVH (10). The theory also proposes neurochemical modulation of interhemispheric STG connectivity by glutamate (Glu) and gamma-aminobutyric acid (GABA), the brain's main excitatory and inhibitory neurotransmitters, respectively. Glu and GABA concentrations are altered in schizophrenia patients compared to healthy control subjects (11, 12). In addition, altered Glu concentrations have specifically been associated with AVH (13–15). Glu and GABA have also been linked to AVH in studies using ketamine, which blocks Glu binding to GABAergic neurons. Administration of ketamine exacerbates psychotic symptoms such as hallucinations in schizophrenia patients (16, 17) and induces schizophrenia-like symptoms in healthy subjects (16, 18–20). Interestingly, ketamine-induced AVH have also been found to be related to increased interhemispheric STG gamma-band connectivity (20), which arises from synchronous firing of GABAergic neurons (21). Together, these findings suggest that Glu- and GABA-modulated interhemispheric STG connectivity plays a role in the occurrence of AVH (10). The interhemispheric miscommunication theory proposes that Glu and GABA in the left STG and prefrontal brain regions might be of particular importance, based on reports of AVH-related altered Glu concentrations in those regions (13, 15). Furthermore, the prefrontal cortex is an area of cognitive control which is central to the interplay between different brain regions, and has previously been suggested to modulate left STG functioning in AVH (22).

The current study investigated the relationship between left STG connectivity and Glu and GABA concentrations in a sample of psychosis patients, with a focus on how this relationship might differ between patients with high hallucination severity (high-AVH) and patients with low hallucination severity (low-AVH). Glu and GABA were measured with hydrogen magnetic resonance spectroscopy (^1^H-MRS). Regions of interest for Glu and GABA measurements were the left STG in the temporal lobe and the anterior cingulate cortex (ACC) in the frontal lobe (10, 14, 22). Glu and GABA were hypothesized to modulate interhemispheric STG functional connectivity, as predicted by the interhemispheric miscommunication theory (10). More specifically, we predicted increased Glu concentrations to be associated with increased connectivity, while we predicted increased GABA concentrations to be associated with decreased connectivity, based on the respective excitatory and inhibitory action of these transmitters. Since AVH have previously been associated with an increase in interhemispheric STG connectivity (4, 5, 20), excitatory effects should be more pronounced in high-AVH patients, whereas inhibitory effects should be more pronounced in low-AVH patients.

## Methods

### Subjects

Data were collected from 81 patients with a psychosis diagnosis, predominantly with a schizophrenia spectrum disorder according to the ICD-10 diagnostic manual (F20-F29: Schizophrenia, schizotypal and delusional disorders) (23). Ten of the patients fulfilled the criterion of hallucination proneness and psychosis but were diagnosed with drug-induced psychotic disorder (*n* = 7) or mood disorders with psychotic symptoms (*n* = 3). The majority of patients used antipsychotic medication, of which all used second-generation antipsychotics, with some patients in addition using first-generation antipsychotics (see Table 1 for details). There were no significant differences between high-AVH and low-AVH patients with respect to duration of illness (*p* = 0.689) or defined daily dose (DDD) of antipsychotic medication (*p* = 0.818). Further information on sample characteristics can be found in Table 1. All subjects gave written informed consent to take part in the study prior to participation.

**Table 1:** Demographic and clinical data for the patient sample.

Age		30.92 (11.56)
Gender (m/f)		58/23
Duration of illness		3.63 (6.31)
**PANSS scores**		
	Total	62.28 (17.65)
	Positive	15.24 (5.52)
	Negative	14.91 (5.16)
	General	32.13 (9.73)
**Medication**		
	DDD antipsychotics	1.05 (0.59)
	Antidepressants	10
	Mood stabilizers	3
	Opioids	1
	Benzodiazepines	13
	Anticholinergic	4
	ADHD	1

*Mean values (and SD) are given. For additional medications, the number of patients per respective group is indicated. PANSS, Positive and Negative Syndrome Scale; DDD, Defined Daily Dose of antipsychotic medication*.

### Clinical Data Acquisition

Clinical data were acquired at the Psychiatric Clinic at the Haukeland University Hospital in Bergen, Norway. The study was approved by the Regional Committee for Medical Research Ethics in Western Norway (REK Vest) (REK # 2010/3387) and conducted according to the Declaration of Helsinki. Severity of AVH was assessed with the P3 item of the Positive and Negative Syndrome Scale [PANSS, (24)]. All PANSS raters were trained and certified, and satisfactory inter-rater reliability was documented. Although the PANSS P3 item does not explicitly differentiate between different sensory modalities of hallucinations, auditory hallucinations are by far the most common type of hallucination in psychotic patients (25), typically in the form of hearing voices (26). Furthermore, AVH are also the main focus during the PANSS P3 interview (27). Therefore, the PANSS P3 is a good indicator of AVH.

### MR Data Acquisition

MR data were acquired on a 3T GE Signa HDx MR scanner in the Haukeland University Hospital in Bergen. In the course of the study, the MR scanner was upgraded to Discovery MR750 and the head coil was changed from 8-channel to 32-channel. The scanner/head coil version was included as a regressor of no interest in all statistical analyses. fMRI resting-state data were collected during a 5.3-min eyes-closed scanning session. There were 160 volumes acquisitions, each with 30 slices containing a 0.5 mm gap between slices (voxel size 1.72 × 1.72 × 3 mm) with the following parameters: repetition time (TR)/echo time (TE)/flip angle (FA)/field of view (FOV) 2,000 ms/30 ms/90°/220 mm. In addition, a structural T1-weighted image was acquired using a 3D SPGR sequence (7.42 min) with the following parameters: TR/TE/FA/FOV 7.78 ms/2.94 ms/14°/256 mm (post-upgrade: 6.9 ms/3.0 ms/12°/256 mm), isotropic voxel size of 1 mm^3^.

**Figure 1:**
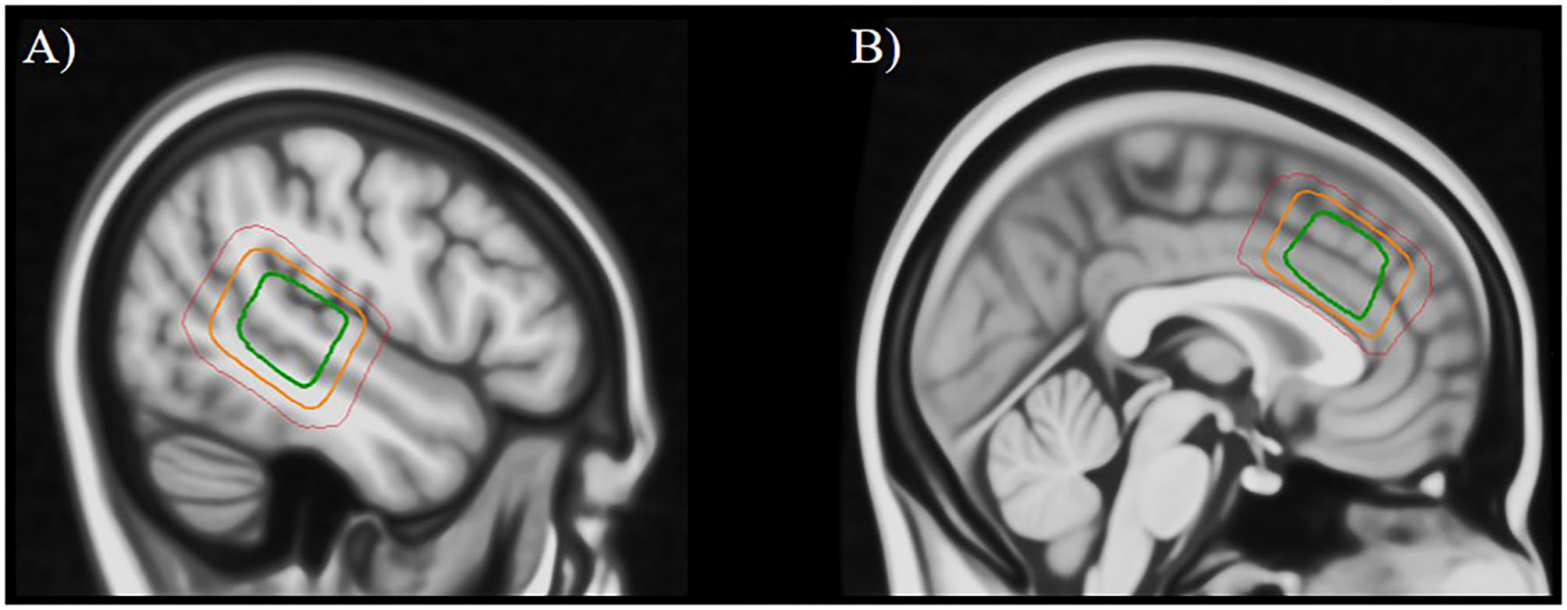
Placement of the MRS voxel in the left superior temporal gyrus **(A)** and the anterior cingulate cortex **(B)**. The three squares indicate the areas that were covered by the individually placed MRS voxels in 95% of subjects (green), 50% of subjects (orange), and 5% of subjects (red).

^1^H-MRS-spectra were obtained from the left STG (voxel size 24 × 40 × 30 mm; Figure 1A) and from the ACC (voxel size 40 × 40 × 25 mm; Figure 1B) by using a single-voxel point-resolved spectroscopy (PRESS) sequence (TE/TR = 35 ms/1,500 ms, 128 repetitions, ~ 4 min), followed by a Mescher-Garwood PRESS (MEGA PRESS) sequence (TE/TR = 68 ms/1,500 ms, 128/192 repetitions pre/post-upgrade with edit pulse at 1.9/7.5 ppm, ~12 min). Unsuppressed water reference spectra (eight repetitions) were acquired automatically after the water-suppressed metabolite spectra. Due to a change in the scanning protocol during the course of the study, only a subsample of patients (*n* = 42) completed the ACC-scan, whereas all patients completed the left STG-scan.

### Data Preprocessing

#### Functional MRI Data

Data were pre-processed in the SPM12 software package (https://www.fil.ion.ucl.ac.uk/spm/). This included realignment of functional volumes for head motion correction, coregistration of the T1 structural image to the mean functional image, normalization of functional data into the MNI (Montreal Neurological Institute) standardized space, and smoothing with a Gaussian kernel of 6 mm FWHM. Data then went through a default denoising procedure implemented in the CONN toolbox (version v.17.f http://www.nitrc.org/projects/conn) where motion realignment parameters (including their first derivatives), as well as time courses from white matter and cerebrospinal fluid were regressed out. Lastly, a band-pass filter of 0.008–0.09 Hz was applied to the data.

#### MR Spectroscopy Data

PRESS and MEGA-PRESS data were processed with the LCModel analysis software, version 6.3-IJ. For details of the processing procedure and quality control of the spectra see (14). Glx (the composite signal of Glu and Gln) and GABA+ (GABA including an unknown macromolecule contribution) levels were used for further analyses. Glx is commonly used as an indicator of Glu concentration levels, since it is a more robust measure than Glu and Gln alone, which can be difficult to separate at 3T. Therefore, the term Glx will hereafter be used when referring to specific results, whereas Glu will be used when discussing general neurochemical mechanisms in a theoretical context. After quality-control, the following number of valid data sets remained: left STG Glx/left STG GABA/ACC Glx/ACC GABA = 81/76/42/41.

### Data Analysis

Seed-based functional connectivity analyses were conducted using the CONN toolbox (https://web.conn-toolbox.org/). A seed region of interest was defined and created for the left STG which covered the MRS voxel location (i.e., voxels that were covered by the left STG MRS voxel in 80% of subjects, transformed into standard space, see Figure 1A). In order to investigate relationships of Glx- and GABA-concentrations with BOLD functional connectivity data, MRS data were included as regressors in seed-to-voxel analyses, together with age, gender and scanner version, which were defined as regressors of no interest. Furthermore, all analyses were repeated with daily defined dose (DDD) of antipsychotic medication as an additional regressor, since antipsychotic medication can affect N-methyl-D-aspartate receptor (NMDA-receptor) functioning and might therefore also affect the Glx and GABA measurements (36,37). However, all results remained substantially unchanged when adding the medication regressor to the analyses. Two groups of patients were compared: patients with high hallucination severity (high-AVH; *n* = 38; PANSS P3 score of ≥3) and patients with low hallucination severity (low-AVH; *n* = 43; P3 score of <3). For the connectivity analyses, a cluster correction procedure was applied with an initial threshold of *p* < 0.001 uncorrected at the single-voxel level and *p* < 0.05 at the cluster-level, FDR-corrected for multiple comparisons. For analyses that did not yield significant results at the pre-set threshold level, the threshold was lowered in order to further explore statistically weaker results.

## Results

### Functional Connectivity and Glx and GABA in the Left STG

#### Glx in the Left STG

**Figure 2:**
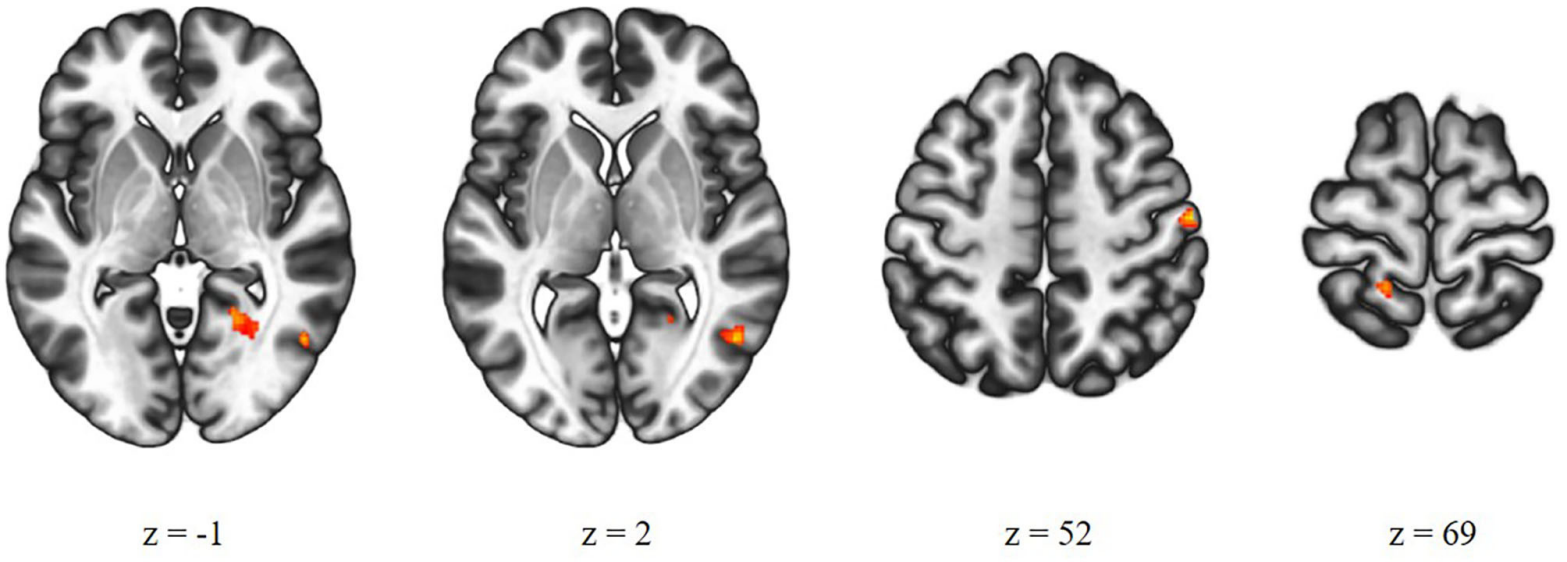
Group comparison of high-AVH patients (*n* = 38) and low-AVH patients (*n* = 43). Glx in left STG had a significantly stronger effect on left STG connectivity with the areas shown in red for high-AVH vs. low-AVH.

There was a significant difference between the high-AVH and the low-AVH group for the relationship between Glx and left STG connectivity with several clusters, primarily bilaterally in the postcentral gyrus and in occipital areas. Investigating Glx effects separately per group showed a negative correlation between Glx and left STG connectivity with the thalamus for the high-AVH group. For the low-AVH group, there was a negative correlation between Glx and connectivity between left STG and left postcentral gyrus. There was no overlap of areas with a significant group difference and those with within-group effects. The results are shown in Table 2 and Figure 2.

**Table 2:** Relationships between Glx concentration in left STG and left STG functional connectivity.

	**Brain area**	**Coordinates**			**Cluster size**	**Beta**	**Voxel-level *p***	**Cluster *p***
**High-AVH – low-AVH**								
	Postcentral gyrus	56	−14	56	83	0.05	<0.001	0.011
	Postcentral gyrus	−15	−43	77	51	0.04	<0.001	0.034
	Lingual gyrus	23	−54	−1	77	0.04	<0.001	0.011
	Lateral/temporo-occipital gyrus	49	−62	2	67	0.05	<0.001	0.015
**Effects within the high-AVH group**								
(*n* = 38)	Thalamus	4	−26	14	52	−0.03	<0.001	0.046
**Effects within the low-AVH group**								
(*n* = 43)	Postcentral gyrus	−41	−29	59	77	−0.03	<0.001	0.023

*The listed clusters showed a group difference in the relationship between Glx and connectivity (high-AVH – low-AVH) or a relationship between Glx and connectivity within one of the groups (effects within high-AVH group and effects within low-AVH group, respectively). Coordinates are given in MNI space. Brain areas are derived from the FSL Harvard-Oxford atlas in Conn. Cluster size is given as the number of voxels*.

#### GABA in the Left STG

There were no significant group differences between the high-AVH and the low-AVH group for the relationship between left STG GABA and left STG connectivity. Furthermore, left STG GABA did not show any significant correlations with left STG connectivity within either of the two groups separately, even when lowering the statistical threshold to voxel-level *p* < 0.01, cluster *p* > 0.05.

### Functional Connectivity and Glx and GABA in the ACC

#### Glx in the ACC

**Figure 3:**
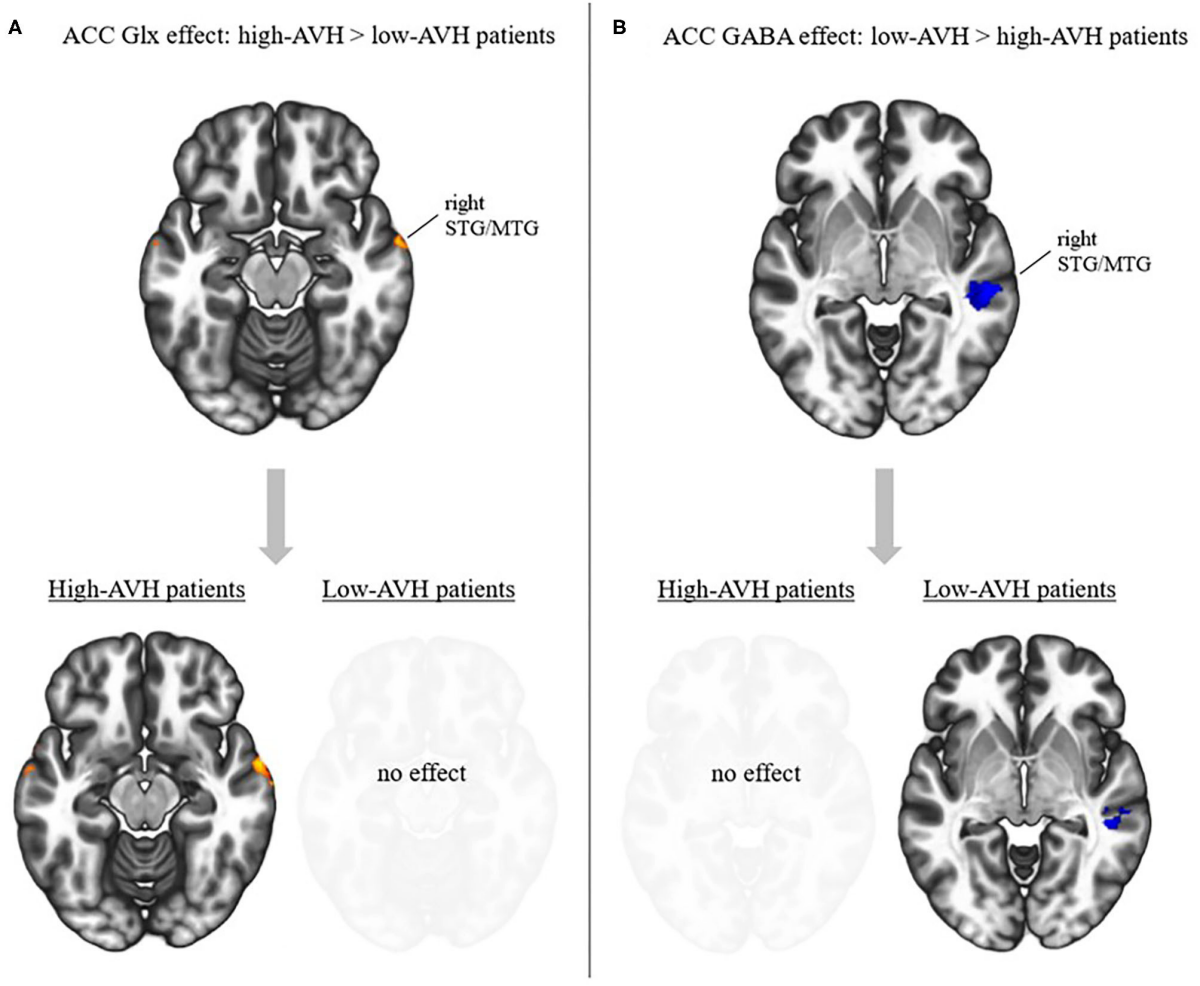
Effects of ACC Glx and GABA on left STG connectivity in relation to AVH. **(A)** Top: Interaction effect between Glx and AVH group: Glx has a stronger effect in high-AVH (*n* = 21) than in low-AVH patients (*n* = 21) on left STG connectivity with the areas shown in red. Bottom: *post-hoc* tests for within-group effects of Glx on connectivity. Higher Glx is associated with increased connectivity between left STG and areas shown in red. **(B)** Top: Interaction effect between GABA and AVH group: GABA has a stronger effect in low-AVH (*n* = 20) than in high-AVH patients (*n* = 21) on left STG connectivity with the areas shown in blue. Bottom: *post-hoc* tests for within-group effects of GABA on connectivity. Higher GABA is associated with decreased connectivity between left STG and areas shown in blue. Slices were taken from MNI coordinates *z* = −15 for Glx effects and *z* = −4 for GABA effects.

**Figure 4:**
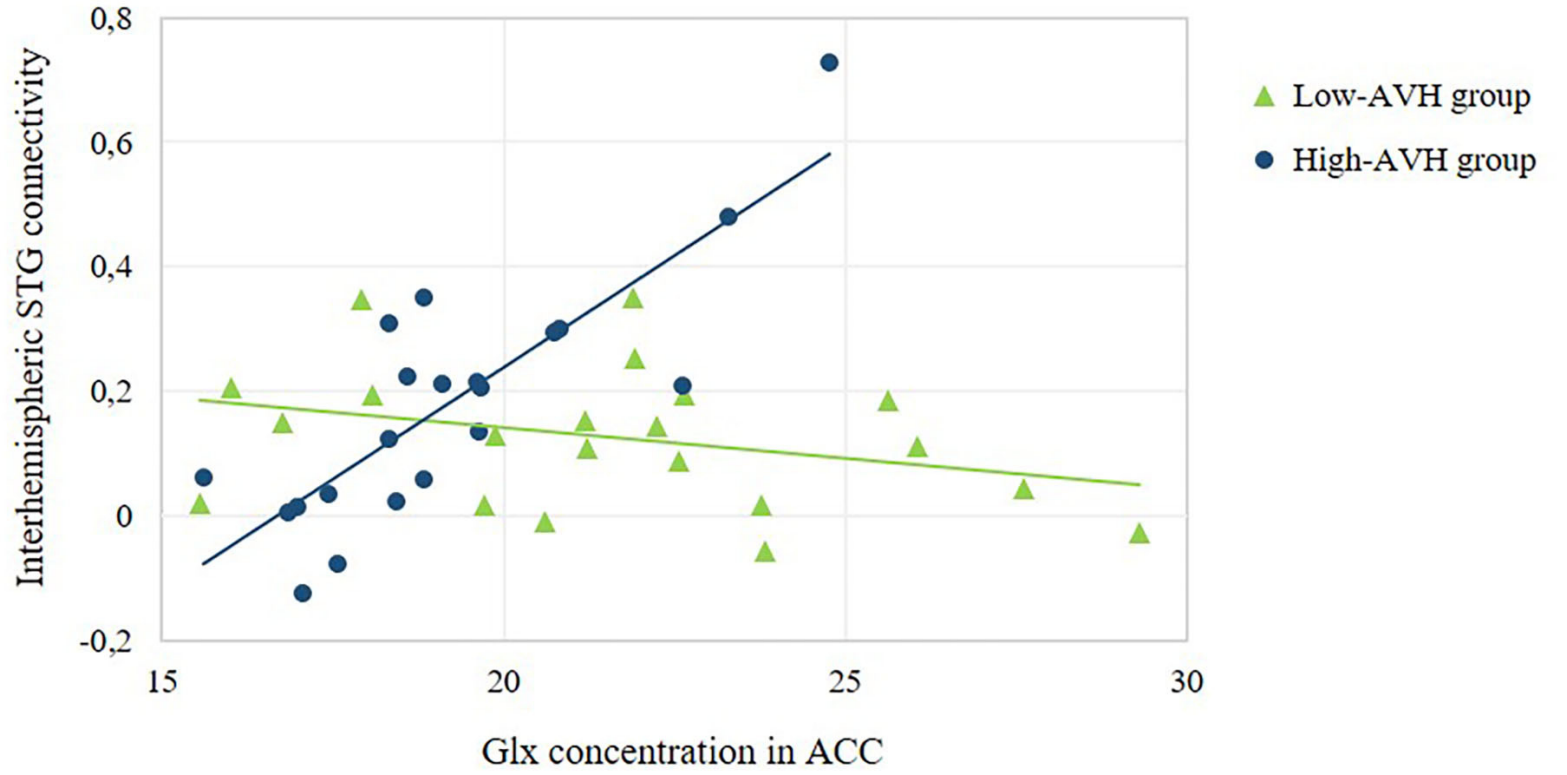
Relationship between ACC Glx concentration and the strength of interhemispheric STG connectivity. The scatterplot offers a numerical depiction of the effect shown in the bottom row of Figure 3A with functional connectivity values extracted from the significant cluster in the right STG/MTG. For low-AVH patients, there was no significant relationship between ACC Glx and left STG connectivity with that cluster, *p* = 0.160. For high-AVH patients, there was a significant positive relationship, *r* = 0.83, *p* = 0.000.

There was a significant Group by Glx interaction, with ACC Glx levels having a significantly stronger effect on left STG connectivity with bilateral STG/MTG and frontal orbital cortex in the high-AVH group compared to the low-AVH group. Within the high-AVH group, there was a positive correlation between Glx and left STG connectivity with left and right STG/MTG, when lowering the voxel-level threshold to voxel-level *p* < 0.01. These STG/MTG clusters overlapped with the areas found significant in the between-group analysis. The results are shown in Table 3 and Figures 3A, 4. For the low-AVH group, there was no significant relationship between Glx and left STG connectivity, irrespective of the statistical thresholding level.

**Table 3:** Relationships between Glx concentration in ACC and left STG functional connectivity.

	**Brain area**	**Coordinates**			**Cluster size**	**Beta**	**Voxel-level *p***	**Cluster *p***
**High-AVH – low-AVH**								
	STG/MTG	−66	−5	2	54	0.11	<0.001	0.027
	STG/MTG	64	3	−4	66	0.09	<0.001	0.021
	Frontal orbital	16	19	−19	49	0.08	<0.001	0.028
**Effects within the high-AVH group**								
(*n* = 21)	STG/MTG	−65	−5	−1	256	0.08	<0.01	0.004
	STG/MTG	63	0	−13	141	0.08	<0.01	0.044
	hippocampus/amygdala	20	−16	−19	157	0.05	<0.01	0.035
	Cerebellum	6	−67	−25	244	−0.02	<0.01	0.004
**Effects within the low-AVH group**								
(*n* = 21)	–		–		–	–	–	–

*The listed clusters showed a group difference in the relationship between Glx and connectivity (high-AVH – low-AVH) or a relationship between Glx and connectivity within one of the groups (effects within high-AVH group and effects within low-AVH group, respectively). Coordinates are given in MNI space. Brain areas are derived from the FSL Harvard-Oxford atlas in Conn. Cluster size is given as the number of voxels*.

#### GABA in the ACC

**Figure 5:**
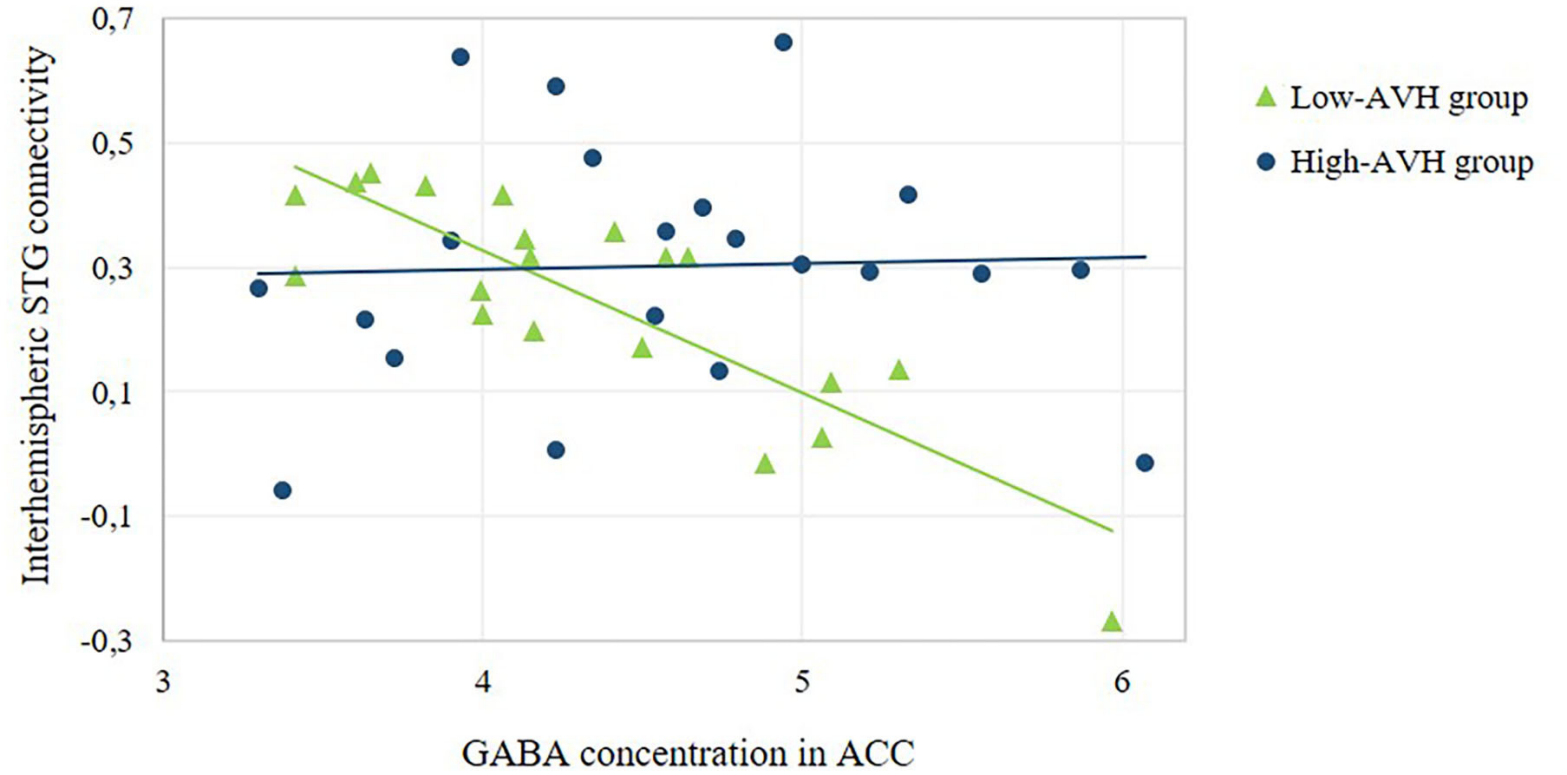
Relationship between ACC GABA concentration and the strength of interhemispheric STG connectivity. The scatterplot offers a numerical depiction of the effect shown in the bottom row of Figure 3B with functional connectivity values extracted from the significant cluster in the right STG/MTG. For high-AVH patients, there was no significant relationship between ACC GABA and left STG connectivity with that cluster, *p* = 0.867. For low-AVH patients, there was a significant negative relationship, *r* = −0.84, *p* = 0.000.

There was a significant Group by GABA interaction, with ACC GABA levels having a stronger effect on left STG connectivity with right STG/MTG in the low-AVH group compared to the high-AVH group. Within the low-AVH group, there was a significant negative correlation between GABA and left STG connectivity with an overlapping right STG/MTG cluster. For the high-AVH group, there was no significant relationship between GABA and STG connectivity. The results are displayed in Table 4 and Figures 3B, 5.

**Table 4:** Relationships between GABA concentration in ACC and left STG functional connectivity.

	**Brain area**	**Coordinates**			**Cluster size**	**Beta**	**Voxel-level *p***	**Cluster *p***
**High-AVH – low-AVH**								
	STG/MTG	57	−28	−4	147	0.31	<0.005	0.027
**Effects within the high-AVH group**								
(*n* = 21)	–		–		–	–	–	–
**Effects within the low-AVH group**								
(*n* = 20)	STG/MTG	47	−33	−4	88	−0.22	<0.001	0.005
	Medial superior frontal gyrus	4	51	44	58	−0.25	<0.001	0.030

*The listed clusters showed a group difference in the relationship between Glx and connectivity (high-AVH – low-AVH) or a relationship between Glx and connectivity within one of the groups (effects within high-AVH group and effects within low-AVH group, respectively). Coordinates are given in MNI space. Brain areas are derived from the FSL Harvard-Oxford atlas in Conn. Cluster size is given as the number of voxels*.

## Discussion

The current study investigated how functional connectivity of the left STG is related to Glx and GABA concentrations in temporal and frontal brain regions and how this relationship is associated with AVH. As hypothesized, Glx and GABA concentration levels in the ACC showed relationships with interhemispheric STG connectivity that were in opposite directions for patients with high vs. low AVH severity. In contrast, Glx and GABA in the left STG did not show any relationships with interhemispheric STG connectivity.

### ACC-Measured Glx and GABA Relationships With Functional Connectivity

The modulation of interhemispheric STG connectivity by Glx and GABA and the AVH-related group differences in this modulation are in line with the interhemispheric miscommunication theory of AVH (10). ACC *Glx* levels were *positively* related to interhemispheric STG connectivity, but only in the high-AVH group. In contrast, ACC *GABA* levels were *negatively* related to interhemispheric STG connectivity, but only in the low-AVH group. The dissociation between Glx and GABA effects is particularly interesting given that the two neurotransmitters are linked, with Glu (the dominant component in Glx measurements) binding to NMDA-receptors on GABAergic neurons as a trigger for the release of GABA. In a previous paper (14), Glx concentrations in the ACC were elevated in low-AVH patients compared to high-AVH patients, and even compared to healthy controls, in a sample that is largely overlapping with that of the current study. This finding was confirmed for the current study's sample. Elevated Glx levels might reflect a compensation mechanism in low-AVH patients to counter schizophrenia-related NMDA-receptor hypo-functioning (13, 28), with elevated Glx concentrations increasing the probability of binding. Such a compensation mechanism might result in sufficient Glu amounts to bind to GABAergic neurons in order to allow for the release of GABA. This would explain the GABA effect on interhemispheric STG connectivity in the low-AVH patients. In contrast, in high-AVH patients, the reduced levels of Glx may prevent a sufficient amount of Glu binding to GABAergic cells, which could explain the absence of a significant GABA effect on connectivity in the high-AVH group. This lack of inhibitory GABA effect to balance the excitatory Glu effect found in the high-AVH group might lead to an increase in interhemispheric STG connectivity in high-AVH patients compared to low-AVH patients, as predicted by the interhemispheric miscommunication theory of AVH (10).

Thus, the current results suggest that it is the interplay between Glu and GABA – i.e., between excitatory and inhibitory effects – that modulates functional connectivity in AVH, and dysfunction in one system may go together with dysfunction in the other system (15). An excitatory/inhibitory imbalance has previously been suggested as a key factor in AVH (14, 29, 30). The current study suggests that one of the mechanisms underlying the relationship between this imbalance and AVH might be altered interhemispheric STG connectivity.

### Left STG-Measured Glx and GABA Relationships With Functional Connectivity

Contrary to our hypothesis, Glx and GABA concentrations in the left STG did not have any AVH-dependent effects on interhemispheric STG connectivity. There were Glx effects on left STG connectivity with occipital and postcentral areas and the thalamus, all of which have been associated with hallucinations (3, 31, 32). However, these findings are difficult to interpret and should be taken with caution, given the lack of overlap of any group differences with within-group effects.

### Limitations of the Study and Directions for Future Research

When interpreting the results of the current study, it should be borne in mind that, for some of the analyses, the sample sizes were relatively small. This may have reduced statistical power and could be the reason that some analyses failed to survive conservative statistical thresholding. These findings should be taken with caution and should be replicated. The fact that the current results were corrected for potential effects of age, gender and medication, should enhance the generalizability to different samples. It is interesting to note that accounting for exposure to antipsychotic medication did not change the results compared to a model where medication was not accounted for. This suggests that antipsychotics did not significantly modulate the relationship between Glx or GABA and left STG connectivity. However, future studies with a pre/post design with fMRI assessment before and after antipsychotic drug exposure might provide more insight into effects of antipsychotics on neurotransmitter concentrations and relationships with functional connectivity.

The current findings on the modulation of interhemispheric STG connectivity by ACC Glx and GABA showed high inner consistency in the sense that group differences were reflected in differential within-group effects in the same brain areas, which strengthens the confidence in these results. The fact that only Glx and GABA in the ACC but not in the left STG showed the expected effects on interhemispheric connectivity is noteworthy and emphasizes the modulatory role of the ACC in AVH. Based on findings of impaired cognitive control in patients with AVH during an auditory task (33) as well as abnormal connectivity between the ACC and the STG (2), it has been suggested that AVH arise from impaired top-down control of prefrontal areas over bottom-up processes in auditory areas (2, 22, 29). The exact role of Glx and GABA in the left STG for brain functioning in AVH remains less clear but could potentially be more strongly related to brain activity, rather than connectivity, given the activation of the left STG during AVH (3, 32, 34).

### Conclusion

Given the rich evidence for AVH-related alterations on the level of brain structure, brain functioning and neurochemistry when studied in isolation, any explanation of AVH will ultimately have to integrate these separate effects at the different levels of explanation (35, 36). The interhemispheric miscommunication theory provides a promising model for such an endeavor since it is based on robust evidence from different levels of explanation, incorporating behavioral, structural and functional abnormalities in interhemispheric STG connectivity. The current study provides an investigation of this model and shows how it is linked to other existing hypotheses about AVH, such as impaired prefrontal top-down control (22) and excitatory/inhibitory imbalances (30). Thereby, the study shows an example of how a combination of different neuroimaging modalities can lead to a more comprehensive understanding of AVH, with possible implications for the development of new therapeutic interventions.

## Data Availability Statement

The raw data supporting the conclusions of this article will be made available by the authors, without undue reservation, to any qualified researcher.

## Ethics Statement

The studies involving human participants were reviewed and approved by Regional Committee for Medical Research Ethics in Western Norway (REK Vest # 2010/3387). The patients/participants provided their written informed consent to participate in this study.

## Disclosure

The authors AC, LE, and KH own shares in the NordicNeuroLab inc. company, which produces some of the add-on equipment used during data acquisition.

## Author Contributions

SW contributed data analysis and interpretation of the data and wrote the first draft of the manuscript. HH contributed to interpretation of the data and wrote parts of the manuscript. AC contributed processing of MRS data. EJ contributed conception and design of the study. RK and E-ML contributed to the data collection. KK organized the database. KH contributed conception and design of the study and interpretation of the data. All authors contributed to manuscript revision.

## Conflict of Interest

The authors declare that the research was conducted in the absence of any commercial or financial relationships that could be construed as a potential conflict of interest.

## Abbreviations

ACC: anterior cingulate cortex
AVH: auditory verbal hallucinations
DDD: defined daily dose
DTI: diffusion tensor imagining
EEG: electroencephalography
fMRI: functional magnetic resonance imaging
GABA: gamma-aminobutyric acid
Glu: glutamate
MRS: magnetic resonance spectroscopy
NMDA: N-methyl-D-aspartate
PANSS: Positive and Negative Syndrome Scale
STG: superior temporal gyrus.
